# Fluctuation of fasting blood glucose in patients who underwent primary or revision total joint arthroplasty: a retrospective review

**DOI:** 10.1186/s13018-020-02029-2

**Published:** 2020-11-05

**Authors:** Yongyu Ye, Baiqi Pan, Minghui Gu, Guoyan Xian, Weishen Chen, Linli Zheng, Ziji Zhang, Puyi Sheng

**Affiliations:** Department of Orthopedic Surgery, The First Affiliated Hospital, Sun Yat-sen University, 58 Zhongshan 2nd Road, Guangzhou, 510080 China

**Keywords:** Total joint arthroplasty, Revision, Fasting blood glucose, Fluctuation, Hyperglycemia

## Abstract

**Background:**

Perioperative hyperglycemia is a risk factor for postoperative complications after total joint arthroplasty (TJA). However, the variability of fasting blood glucose (FBG) after TJA remains unknown. We aimed to assess the fluctuation and extent of elevation of FBG following primary or revision TJA.

**Methods:**

We retrospectively evaluated the medical records of 1788 patients who underwent primary or revision TJA between 2013 and 2018. We examined FBG values collected during 6 days of the perioperative period. The findings for each time point were evaluated with descriptive statistics. Postoperative glycemic variability was assessed by the coefficient of variation (CV).

**Results:**

The final cohort included the medical records of 1480 patients (1417 primary and 63 revision). FBG was highest on postoperative day 1 in the primary and revision groups (*P* < 0.001), which had the highest number of hyperglycemic patients (FBG > 100 mg/dL), with 66.4% and 75.5% in the primary and revision groups, respectively. The CV of diabetics in the primary group, and diabetics and non-diabetics in the revision group, was higher than that of non-diabetics in the primary group.

**Conclusion:**

Postoperative day 1 showed the highest FBG levels and proportion of patients with hyperglycemia in the perioperative period. Primary group diabetics, and revision group diabetics and non-diabetics, had higher postoperative fluctuation of FBG than primary group non-diabetics. Frequent FBG monitoring may therefore be warranted in diabetic patients undergoing TJA, and all patients undergoing revision TJA.

## Background

Total joint arthroplasty (TJA) is a surgical procedure to treat late-stage osteoarthritis and improve the quality of life [1]. However, more complications are being recorded than previously, due to the increased number of TJA surgeries [2, 3]. Risk factors for postoperative complications, including age, obesity, malnutrition, prior infection, and hyperglycemia, were demonstrated by several studies [4–6]. Nonetheless, translation of these factors into clinical practice to avoid complications and reach promising outcomes has been addressed critically important [7–10]. For example, correction of malnutrition and controlling the blood glucose within normal levels would have a great impact on minimizing postoperative complications [11, 12].

Recent studies have confirmed hyperglycemia as a significant risk factor for postoperative complications in TJA, independent of diabetes mellitus (DM) status [4, 6, 13]. Timely recognition of fluctuation of glucose levels and maintaining glycemic control is crucial for reducing undesired complications [6]. Kheir et al. noted a linear relationship between postoperative blood glucose and periprosthetic joint infection (PJI) [4], while Shohat et al. demonstrated that higher postoperative glucose variability was associated with increased rates of complications [6]. Varady et al. suggested that 9 pm on the night of TJA surgery was the most sensitive time within 24 h for detecting hyperglycemia in both diabetic and non-diabetic patients [14]. Though some researchers have demonstrated the variability of glucose levels in patients undergoing TJA and acute fluctuation of blood glucose within 24 h following TJA, an exact conclusion regarding fasting blood glucose (FBG) fluctuation and the extent to which FBG may become elevated postoperatively in TJA patients remains unknown.

Therefore, we investigated the variability and distribution of FBG during 6 days postoperatively in patients undergoing primary or revision TJA to explore the differences and fluctuation of FBG, which may aid clinicians in controlling FBG in various aspects of clinical practice.

## Materials and methods

### Selection criteria and data review

Following approval by our institutional review board, this retrospective study reviewed 1788 medical records of patients who were admitted to the orthopedic department of our hospital between October 2013 and November 2018. We included patients who underwent total knee arthroplasty (TKA) or total hip arthroplasty (THA) as either a primary or revision surgical procedure. The exclusion criteria were as follows: (1) patients with underlying inflammatory conditions, e.g., rheumatoid arthritis and ankylosing spondylitis, as this would impact blood glucose levels; (2) patients with malignancy, as evidence showed patients with malignancy had higher blood glucose levels [15]; (3) no previous surgical record; (4) lack of FBG data within 6 days postoperatively. Finally, the medical records of 1417 primary and 63 revision TJA patients were selected for the study. Demographic information (age, sex, type of procedure, date of admission, date of discharge), comorbidities (malignancy, rheumatoid arthritis, ankylosing spondylitis, DM, and hypertension), operative details (type of anesthesia, date of surgery, start time, end time, and duration of surgery), and reasons for surgery (PJI, periprosthetic osteolysis, aseptic loosening, periprosthetic fracture, dislocation, and surgical site infection [SSI]) were extracted from the records. The definitions of SSI and PJI were based on the Centers for Disease Control (CDC) definition and the Musculoskeletal Infection Society criteria [16, 17]. The medial parapatellar approach was used for TKA, and the posterior lateral approach was used for THA. The requirement for informed consent was waived due to the retrospective design of our study.

### Laboratory evaluation and management protocol

Routine blood tests, including basic metabolic panel, were ordered preoperatively for all patients admitted for arthroplasty. FBG values were based on serum glucose levels in the basic metabolic panel preoperatively and during the 6 days postoperatively. All samples were drawn at approximately 7 am after patients had fasted overnight for a minimum of 8 h. The diagnosis of DM was based on past medical history or the American Diabetes Association (ADA) criteria regarding the blood glucose after admission. Patients with DM were given diabetic meals, and those without were given standard meals, from the hospital canteen. All patients were on restricted solids and liquids for at least 8 h before surgery. Either general or spinal anesthesia was performed. Postoperatively, most patients received an analgesic pump. All patients were given lactated Ringer’s solution or glucose saline intravenously within 6 h following surgery. In case of hyperglycemia resulting from the infusion of glucose, supplementary insulin was added to the glucose saline for diabetic patients. Patients with DM were initiated on sliding-scale insulin therapy or antidiabetic drugs. Fixed-amount carbohydrate meals were provided to patients with DM at each meal from the hospital canteen. Patients with DM underwent regular fingertip glucose level monitoring and adjustment of oral medications or insulin according to their glucose levels. Non-steroidal anti-inflammatory drugs such as loxoprofen or celecoxib were used to relieve pain postoperatively. Routine blood tests postoperative were performed for all patients. Ordering of blood tests on other days was determined by the doctors in charge and the general status of patients. After discharge, patients underwent regular follow-up.

### Classification of FBG

We classified FBG levels into several categories to identify the hyperglycemic state of each patient at each time point, based on previous studies [4, 13, 14, 18–22]. According to the definition from the ADA and evidence published by Varady et al. [14, 18], the normal status was defined as FBG < 100 mg/dL. FBG between 100 mg/dL and 126 mg/dL was defined as elevated blood glucose. The other three categories for FBG used to define hyperglycemia were as follows: strict > 126 mg/dL, intermediate > 137 mg/dL, and lenient > 180 mg/dL. Postoperative glycemic variability was evaluated by calculating the coefficient of variation (CV), which is the ratio of the standard deviation to the mean FBG [23, 24].

### Statistical analysis

For the demographic features, age differences were analyzed using the independent *t* test, and categorical variables were calculated using the chi-square test. The Mann-Whitney *U* test was applied for divergence in glycemic status. The measures of FBG (mean, standard deviation, median, range, and hyperglycemic rate) were calculated at each time point. The variability and fluctuation of FBG were revealed by boxplot and CV. To evaluate the differences between each pair of time points, we used the Kruskal-Wallis *H* test for comparisons, and the *P* value was adjusted by Bonferroni analysis. A *P* value < 0.05 was considered statistically significant. All analyses were performed using SPSS (Statistical Package for the Social Sciences) version 22 (IBM Corporation, Armonk, New York).

## Results

### Demographic features and general glycemic status

Medical records of 1480 patients (1417 primary and 63 revision) were studied. There were no significant differences in age and sex ratio between the two groups (*P* = 0.17 and *P* = 0.10, respectively). In the revision group, the number of patients with hip problems was significantly higher than that in the primary group (*P* < 0.001). For DM, hypertension, and anesthesia, no significant differences were revealed between the primary and revision groups. For the glycemic status of FBG, significant differences were noted between primary and revision patients postoperatively (*P* = 0.01) (Table 1).

**Table 1:** Demographic features and glycemic status of patients who underwent total joint arthroplasty

Group and variable	Primary	Revision	*P* value
No. of patients	1417	63	
Age^a^	63.3 ± 12.3	65.5 ± 11.1	0.17^c^
Sex^b^			0.10^d^
Female	1012 (71.4%)	39 (61.9%)	
Male	405 (28.6%)	24 (38.1%)	
Joint ^b^			< 0.001^d^
Hip	604 (42.6%)	53 (84.1%)	
Knee	813 (57.4%)	10 (15.9%)	
Diabetes mellitus^b^			0.17^d^
Diabetic	184 (13.0%)	12 (19.0%)	
Non-diabetic	1233 (87.0%)	51 (81.0%)	
Blood pressure^b^			0.23^d^
Hypertension	580 (40.9%)	42 (66.7%)	
Non-hypertension	837 (59.1%)	21 (33.3%)	
Anaesthesia^b^			0.28^d^
Spinal	1032 (72.8%)	43 (68.3%)	
General	385 (27.2%)	20 (31.7%)	
Glycemic status (mg/dL)^a^			
Pre-operation	93 ± 26	94 ± 28	0.81^e^
Post-operation (within 6 days)	109 ± 32	121 ± 45	0.01^e^
Total	103 ± 31	111 ± 42	0.55^e^

*TJA* total joint arthroplasty

^a^Data are presented as the mean ± standard deviation

^b^Data are presented as the number (percentage) of patients

^c^*P* value was calculated by the independent *t* test

^d^*P* value was calculated by the chi-square test

^e^*P* value was calculated by the Mann-Whitney *U* test. *P* < 0.05 indicates a significant difference between groups

### The fluctuation of perioperative FBG

#### Primary total joint arthroplasty patients

**Fig. 1:**
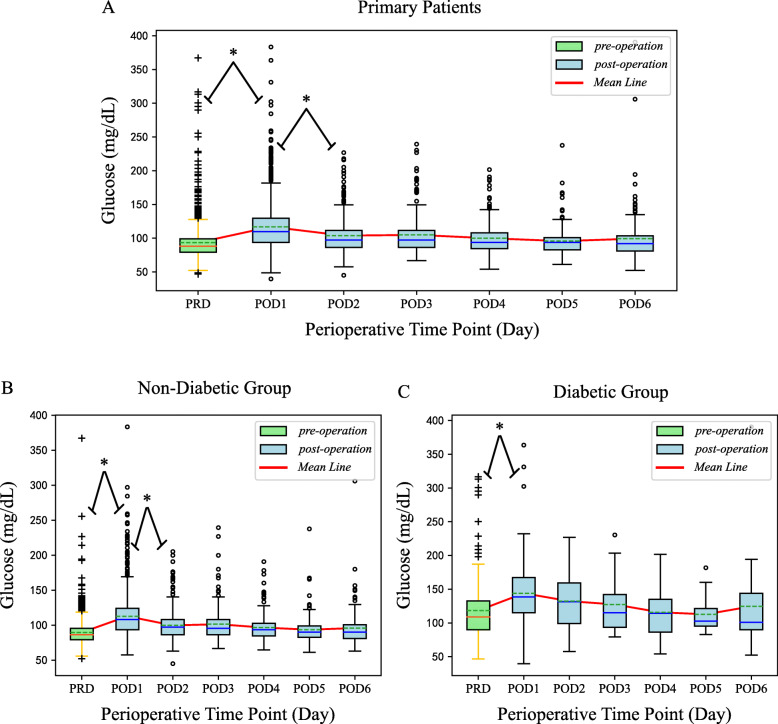
**a-c** Boxplots of FBG for each time point across patients with primary total joint arthroplasty. **a** The fluctuation of FBG for each time point in patients with primary total joint arthroplasty. **b**, **c** The fluctuation of FBG for each time point in patients with primary total joint arthroplasty grouped by diabetic status. The asterisks indicate a significant difference between groups. The *P* values were calculated using the Kruskal-Wallis *H* test and corrected by Bonferroni analysis. Solid lines within the box indicate median, top, and bottom lines of the box equal interquartile range (IQR), whiskers indicate values within 1.5 IQR of the top or bottom of the box, and circle or plus symbols represent outliers. Dashed lines within the box indicate mean and the red line represents the fluctuation of the mean of FBG. PRD indicates preoperative day. POD indicates postoperative day, and the number represents the day after surgery

Among all patients (diabetic and non-diabetic together) or non-diabetic group with primary TJA, the mean glucose levels at POD1 (postoperative day 1) were significantly higher than that at all other time points (*P* < 0.001). There were no significant differences in FBG between PODs 2 and 3, PODs 3 and 4, PODs 4 and 5, or PODs 5 and 6. From PODs 1 to 6, FBG gradually declined and became stable (CVs of all patients and non-diabetic patients: 7.14% and 6.85% respectively). Further examination of the primary group revealed that FBG at POD1 in diabetics was only significantly higher than that measured preoperatively (*P* < 0.001), and the variability of FBG was higher in diabetics within the primary group (CV%, 9.02) (Table 2, Fig. 1).

**Table 2:** Perioperative glucose levels of the primary total joint arthroplasty population

Day	Cases (*n*)	Glucose level (mg/dL)								CV (%)^b^
		Mean ± SD	Median	Range	Normal^a^ (< 100 mg/dL)	Elevated^a^ (100-126 mg/dL)	Strict^a^ (> 126 mg/dL)	Intermediate^a^ (> 137 mg/dL)	Lenient^a^ (> 180 mg/dL)	
Total										
PRD	1463	93 ± 26	88	47-367	1133 (77.4%)	229 (15.7%)	101 (6.9%)	69 (4.7%)	20 (1.4%)	
POD1	1158	117 ± 35	110	40-383	388 (33.5%)	429 (37.0%)	341 (29.4%)	228 (19.7%)	58 (5.0%)	7.14
POD2	470	104 ± 26	97	45-227	255 (54.3%)	154 (32.8%)	61 (13.0%)	45 (9.6%)	10 (2.1%)	
POD3	217	105 ± 29	97	67-239	119 (54.8%)	66 (30.4%)	32 (14.7%)	22 (10.1%)	9 (4.1%)	
POD4	205	100 ± 24	94	54-202	132 (64.4%)	46 (22.4%)	27 (13.2%)	18 (8.8%)	4 (2.0%)	
POD5	177	96 ± 21	94	61-238	126 (71.2%)	40 (22.6%)	11 (6.2%)	7 (4.0%)	2 (1.1%)	
POD6	143	99 ± 38	92	52-391	99 (69.2%)	26 (18.2%)	18 (12.6%)	13 (9.1%)	4 (2.8%)	
Diabetic										
PRD	194	118 ± 45	109	47-317	81 (41.8%)	53 (27.3%)	60 (30.9%)	45 (23.2%)	14 (7.2%)	
POD1	153	144 ± 47	139	40-364	23 (15.0%)	35 (22.9%)	95 (62.1%)	77 (50.3%)	26 (17.0%)	9.02
POD2	59	132 ± 40	131	58-227	15 (25.4%)	14 (23.7%)	30 (50.8%)	26 (44.1%)	7 (11.9%)	
POD3	29	127 ± 41	115	79-230	10 (34.5%)	6 (20.7%)	13 (44.8%)	8 (27.6%)	5 (17.2%)	
POD4	36	116 ± 35	114	54-202	13 (36.1%)	8 (22.2%)	15 (41.7%)	9 (25.0%)	3 (8.3%)	
POD5	19	113 ± 26	103	83-182	6 (31.6%)	9 (47.4%)	4 (21.1%)	3 (15.8%)	1 (5.3%)	
POD6	17	125 ± 77	101	52-391	8 (47.1%)	4 (23.5%)	5 (29.4%)	5 (29.4%)	2 (11.8%)	
Non-diabetic										
PRD	1269	90 ± 18	86	52-367	1052 (82.9%)	176 (13.9%)	41 (3.2%)	24 (1.9%)	6 (0.5%)	
POD1	1005	113 ± 30	108	58-383	365 (36.3%)	394 (39.2%)	246 (24.5%)	151 (15.0%)	32 (3.2%)	6.85
POD2	411	100 ± 21	97	45-205	240 (58.4%)	140 (34.1%)	31 (7.5%)	19 (4.6%)	3 (0.7%)	
POD3	188	101 ± 24	95	67-239	109 (58.0%)	60 (31.9%)	19 (10.1%)	14 (7.4%)	4 (2.1%)	
POD4	169	97 ± 20	94	65-191	119 (70.4%)	38 (22.5%)	12 (7.1%)	9 (5.3%)	1 (0.6%)	
POD5	158	94 ± 20	90	61-238	120 (75.9%)	31 (19.6%)	7 (4.4%)	4 (2.5%)	1 (0.6%)	
POD6	126	96 ± 28	90	63-306	91 (72.2%)	22 (17.5%)	13 (10.3%)	8 (6.3%)	2 (1.6%)	

*PRD* pre-operative day, *POD* post-operative day, *SD* standard deviation

^a^Data are presented as the number (percentage) of measurement

^b^Post-operative glycemic variability was assessed using a coefficient of variation (the ratio of the standard deviation to the mean glucose level)

We further classified hyperglycemia into four groups. POD1 had the highest number of hyperglycemic patients (66.4% for FBG > 100 mg/dL, 37.0% for FBG of 100-126 mg/dL, and 29.4% for FBG > 126 mg/dL). When grouped by diabetic status, 63.7% of non-diabetic and 85.0% of diabetic patients were hyperglycemic (> 100 mg/dL) at POD1 (Table 2).

#### Revision surgery patients

**Fig. 2:**
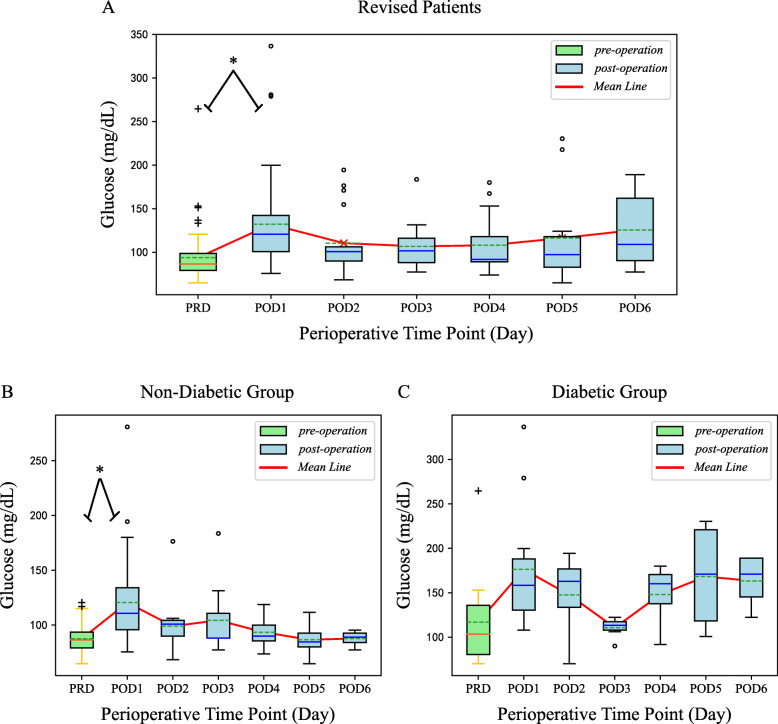
**a-c** Boxplots of FBG for each time point across patients with revision surgery. **a** The fluctuation of FBG for each time point in patients with revision surgery. **b**, **c** The fluctuation of FBG for each time point in patients with revision surgery grouped by diabetic status. The asterisks indicate a significant difference between groups. The *P* values were calculated using the Kruskal-Wallis *H* test and corrected by Bonferroni analysis

In the revision group, FBG at POD1 in all patients (diabetic and non-diabetic together) or non-diabetic group was significantly higher than at the PRDs (preoperative days) (*P* < 0.001). There were no significant differences in FBG between PODs 2 and 3, PODs 3 and 4, PODs 4 and 5, or PODs 5 and 6. The CVs of all patients and non-diabetic patients were 8.82% and 12.83% postoperatively, and FBG displayed large fluctuation. However, compared to the non-diabetic group, there were no significant differences in FBG between each time point in the diabetic group, and the variability of FBG was higher (CV%, 15.31) (Table 3, Fig. 2).

**Table 3:** Perioperative glucose levels of patients with revision surgery

Day	Cases (*n*)	Glucose level (mg/dL)								CV (%)^b^
		Mean ± SD	Median	Range	Normal^a^ (< 100 mg/dL)	Elevated^a^ (100-126 mg/dL)	Strict^a^ (> 126 mg/dL)	Intermediate^a^ (> 137 mg/dL)	Lenient^a^ (> 180 mg/dL)	
Total										
PRD	66	94 ± 28	86	65-265	50 (75.8%)	11 (16.7%)	5 (7.6%)	3 (4.5%)	1 (1.5%)	
POD1	53	132 ± 50	121	76-337	13 (24.5%)	14 (26.4%)	26 (49.1%)	16 (30.2%)	6 (11.3%)	8.82
POD2	17	110 ± 39	101	68-194	7 (41.2%)	6 (35.3%)	4 (23.5%)	4 (23.5%)	1 (5.9%)	
POD3	16	107 ± 26	102	77-184	8 (50.0%)	6 (37.5%)	2 (12.5%)	1 (6.2%)	1 (6.2%)	
POD4	15	108 ± 33	92	74-180	9 (60.0%)	3 (20.0%)	3 (20.0%)	3 (20.0%)	1 (6.7%)	
POD5	11	116 ± 56	97	65-230	6 (54.5%)	3 (27.3%)	2 (18.2%)	2 (18.2%)	2 (18.2%)	
POD6	8	126 ± 46	109	77-189	4 (50.0%)	1 (12.5%)	3 (37.5%)	3 (37.5%)	2 (25.0%)	
Diabetic										
PRD	14	117 ± 51	104	70-265	6 (42.9%)	3 (21.4%)	5 (35.7%)	3 (21.4%)	1 (7.1%)	
POD1	11	176 ± 72	158	108-337	0 (0.0%)	2 (18.2%)	9 (81.8%)	7 (63.6%)	3 (27.3%)	15.31
POD2	4	148 ± 54	163	70-194	1 (25.0%)	0 (0.0%)	3 (75.0%)	3 (75.0%)	1 (25.0%)	
POD3	6	111 ± 12	113	90-122	1 (16.7%)	5 (83.3%)	0 (0.0%)	0 (0.0%)	0 (0.0%)	
POD4	4	148 ± 39	160	92-180	1 (25.0%)	0 (0.0%)	3 (75.0%)	3 (75.0%)	1 (25.0%)	
POD5	4	168 ± 65	171	101-230	0 (0.0%)	2 (50.0%)	2 (50.0%)	2 (50.0%)	2 (50.0%)	
POD6	4	163 ± 32	171	122-189	0 (0.0%)	1 (25.0%)	3 (75.0%)	3 (75.0%)	2 (50.0%)	
Non-diabetic										
PRD	52	88 ± 12	86	65-121	44 (84.6%)	8 (15.4%)	0 (0.0%)	0 (0.0%)	0 (0.0%)	
POD1	42	121 ± 36	111	76-281	13 (31.0%)	12 (28.6%)	17 (40.5%)	9 (21.4%)	3 (7.1%)	12.83
POD2	13	99 ± 27	101	68-176	6 (46.2%)	6 (46.2%)	1 (7.7%)	1 (7.7%)	0 (0.0%)	
POD3	10	104 ± 32	88	77-184	7 (70.0%)	1 (10.0%)	2 (20.0%)	1 (10.0%)	1 (10.0%)	
POD4	11	93 ± 15	90	74-119	8 (72.7%)	3 (27.3%)	0 (0.0%)	0 (0.0%)	0 (0.0%)	
POD5	7	87 ± 15	85	65-112	6 (85.7%)	1 (14.3%)	0 (0.0%)	0 (0.0%)	0 (0.0%)	
POD6	4	88 ± 8	89	77-95	4 (100.0%)	0 (0.0%)	0 (0.0%)	0 (0.0%)	0 (0.0%)	

*PRD* pre-operative day, *POD* post-operative day, *SD* standard deviation

^a^Data are presented as the number (percentage) of measurement

^b^Post-operative glycemic variability was assessed using a coefficient of variation (the ratio of the standard deviation to the mean glucose level)

The highest number of hyperglycemic (> 100 mg/dL) patients in the revision group was at POD1, which was far higher than that on the other days. When stratified by diabetic status, 69.1% of non-diabetic and 100% of diabetic patients were hyperglycemic (> 100 mg/dL) at POD1 (Table 3).

## Discussion

The clear association between perioperative hyperglycemia and postoperative complications (for example, PJI) after TJA has been established by previous studies [4, 6, 25–27]. However, the fluctuation and extent of elevation of FBG within 1 week after TJA remains uncertain. In our study, we studied the FBG monitoring records through 6 days following TJA. We found that POD1 had the highest FBG level and the most hyperglycemic patients perioperatively, suggesting that investigating FBG at this time point may be warranted. Additionally, compared to non-diabetic patients in the primary group, the fluctuation of FBG was greater in patients with DM within the primary group and diabetic and non-diabetic patients in the revision group, indicating that frequent perioperative FBG assessment was useful for those patients.

Hyperglycemia, which can even occur in non-diabetics, was considered to be a risk factor for postoperative complications [4, 14, 20]. Notably, evidence shows that monitoring blood glucose levels and assessing glucose variability are effective in predicting complications [6]. Two studies showed that early postoperative glucose control could be a reducing factor in nosocomial infections [28, 29]. Specifically, Varady et al. examined the blood glucose values within POD1 in patients who underwent TJA and found that 9 pm on the night of surgery showed the highest number of hyperglycemic patients, suggesting that this time point may be the most sensitive for detecting hyperglycemia in both diabetic and non-diabetic patients [14]. However, the above studies lacked multiple consecutive monitoring of postoperative FBG, and there is no consensus regarding how FBG varies perioperatively. While their data were limited to 24 h after surgery, our study included not only the observation of the glucose fluctuation in PRDs but also for 6 days postoperatively. In our study, the peak FBG appeared in the first 24 h after surgery in the primary and revision groups. Our findings suggest that the FBG for TJA patients remains within normal limits before surgery and increases substantially within the first day of TJA, and then postoperative FBG decreases mildly and tends toward normal in the non-diabetic patients within the primary group. Various factors could contribute to the elevation of blood glucose levels postoperatively, including medications, physiologic stress response, and hormones [4, 30]. More importantly, the episodes of hyperglycemia would provide an optimal medium for bacterial growth, alter the immune response, and subsequently impair the capability of the host to battle infection, making patients more susceptible to infection postoperatively [31, 32]. Regardless of the etiology and pathophysiology of postoperative hyperglycemia, several studies have demonstrated the association between hyperglycemia in POD1 and postoperative complications [4, 19, 21], which was in line with our findings. Kheir et al. revealed that blood glucose levels in POD1 were significantly associated with PJI, and the risk of PJI increased linearly when blood glucose levels > 115 mg/dL. Furthermore, they set a glucose level of 137 mg/dL as the optimal threshold to reduce the likelihood of PJI [4]. Another study demonstrated that significantly higher blood glucose levels in POD1 were observed in infected patients. Interestingly, non-diabetic patients with blood glucose values of > 140 mg/dL in POD1 were more than 3 times likely to develop PJI than those with equivalent glucose levels in the DM group [19]. However, further studies are needed to monitor the blood glucose levels after TJA in a continuous manner, aiming to determine the most sensitive time point in detecting hyperglycemia.

On the other hand, previous studies have suggested that clinicians should gain better control of glucose levels below the cutoff values to minimize the risk of postoperative complications [4, 13, 16, 19, 21]. CDC guidelines, published in 2017, stipulate that it is better to maintain blood glucose levels at less than 200 mg/dL for all patients during surgery, regardless of diabetic status [16]. Kwon et al. studied the relationship between perioperative hyperglycemia and outcomes in general surgery. They revealed that glucose levels maintained below 130 mg/dL had promising outcomes [21]. Kheir et al. found that hyperglycemia was associated with PJI, with an optimal cutoff of 137 mg/dL [4]. Mraovic et al. investigated the association between hyperglycemia and infection after TJA and found that postoperative blood glucose values of > 140 mg/dL doubled the risk of infection in patients with TJA [19]. Kremers et al. demonstrated a significantly higher risk of PJI among patients with perioperative hyperglycemia (blood glucose value > 180 mg/dL) [13]. In our study, we classified hyperglycemia into different categories. For both the primary and revision groups, the FBG increased significantly at POD1, and the total number of hyperglycemic patients was the highest at POD1 among all measured days, indicating that POD1 was the most critical and sensitive day for clinicians to monitor FBG. At POD1, FBG in many patients was beyond the cutoff values, which suggested an increased risk of postoperative complications based on previous evidence [4, 13, 16, 19, 21]. Notably, controlling and maintaining glucose levels under thresholds was likely to reduce complications, which was supported by previous studies [21, 33]. Gallagher et al. pointed out that subcutaneous insulin intervention was both effective and safe for the management of postoperative hyperglycemia in TJA patients with or without DM, and the rate of developing PJI was remarkedly low by controlling hyperglycemia in a timely manner [33]. Besides investigations in the arthroplasty literature, a study regarding the general surgery as well verified the evidence that implementing insulin to lower blood glucose levels in hyperglycemic patients postoperatively ameliorates the risk of postoperative complications [21]. Therefore, clinicians should identify hyperglycemic patients by earlier monitoring after TJA and be aware that well controlling of blood glucose at POD1 can minimize the duration that the patient spends in a hyperglycemic state, which could decrease postoperative complications and improve clinical outcomes.

Understanding blood glucose variability could help doctors monitor and maintain blood glucose at stable levels [6]. Maeda et al. performed continuous glucose monitoring analysis in 20 patients who underwent THA or TKA and found that higher blood glucose levels and larger fluctuations were detected postoperatively, especially until POD2 [34]. Shohat et al. focused on the associations between hyperglycemia and adverse outcomes in patients who underwent orthopedic surgery and TJA, and they demonstrated that higher glucose variability postoperatively was associated with increased rate of complications [6, 25]. However, the extent of FBG fluctuation remains unknown. In our study, from PODs 1 to 6, the FBG gradually decreased and became stable in non-diabetic patients in the primary group. For diabetics in the primary group and diabetics and non-diabetics in the revision group, the FBG varied dramatically in the postoperative period. Greater fluctuation of FBG was observed in patients with DM and those in the revision group. Hence, the FBG of patients with DM and those undergoing revision surgery should be carefully monitored to ensure prompt detection and control of hyperglycemia.

To our knowledge, our study is the first to continuously monitor and examine the fluctuation of FBG over almost a week after TJA. However, there are some limitations to this study. First, not all confounding variables that could impact blood glucose, such as the body mass index, were considered. Second, this is a retrospective study, and not all the patients had FBG measured at each time point. Postprandial glucose and HbA1c could not be studied due to the lack of relevant extractable medical information. Third, compared to the primary group, the number of patients in the revision group was relatively small. Future studies assessing a larger cohort of revision patients and measuring glucose values at fixed intervals would be valuable to gain further insight and better patient representation.

## Conclusions

Our study highlights that POD1 had the highest FBG levels and proportion of patients with hyperglycemia perioperatively. Greater attention should be paid to frequent measurement of FBG in patients with TJA after surgery, especially for patients who have DM or those who undergo revision surgery because the fluctuation of FBG was higher among such patients in our study. Clinicians should be aware of the incidence of perioperative hyperglycemia during fasting, and strategies to regulate glucose levels must be developed and implemented to avoid postoperative complications. Prospective multicenter studies are needed to further elucidate the value of detecting and controlling postoperative hyperglycemia, and thus diminish postoperative complications in patients with TJA.

## Data Availability

The datasets used during the current study are available from the corresponding author on reasonable request.

## Abbreviations

ADA: American Diabetes Association
CDC: Centers for Disease Control
DM: Diabetes mellitus
PJI: Periprosthetic joint infection
SSI: Surgical site infection
THA: Total hip arthroplasty
TKA: Total knee arthroplasty
TJA: Total joint arthroplasty
POD: Postoperative day
PRD: Preoperative day
CV: Coefficient of variation
